# HIV positive asylum seekers receiving the order to leave the Belgian territory

**DOI:** 10.7448/IAS.17.4.19592

**Published:** 2014-11-02

**Authors:** Remy Demeester, Jean-Claude Legrand

**Affiliations:** Infectious Diseases, University Hospital of Charleroi, Charleroi, Belgium

## Abstract

**Introduction:**

In a human rights based approach, the Parliamentary Assembly of the Council of Europe has recently released a resolution about migrants and refugees and the fight against HIV [1]. It states that “an HIV positive migrant should never be expelled when it is clear that he will not receive adequate health care and assistance in the country to which he is being sent back. To do otherwise would amount to a death sentence for that person.” Nevertheless, in Belgium, for the last 2 years, none of the HIV-infected migrants in care in the AIDS Reference Centers (ARC) received the right to stay in Belgium for medical reasons.

**Methods:**

We identified all HIV-infected asylum seekers in care between 1 July 2012 and 1 July 2014 in the ARC of Charleroi, Belgium, and we analyzed their medical and social files.

**Results:**

Among the 302 patients in active follow up in our ARC, 45 HIV positive asylum seekers were in care during the last 2 years. Male/female ratio was 0/96. Mean age was 35 years. Countries of origin and reasons for migration are detailed in the Table 1. 18% (8/45) knew their seropositivity before arriving in Europe. All the patients introduced an asylum request, 29 (64%) have received a negative answer and an order to leave the territory, 4 (9%) were regularized for non-medical reasons (see Table 1), 4 (9%) are waiting for an answer and for 8 (18%) outcome is unknown due to lost follow up (LFU). 31 (69%) patients have also introduced a request to stay for medical reasons: 18 (58%) have received a refusal, 7 (23%) are still waiting for an answer, and 6 (19%) are LFU. Only 23 (51%) patients are still in care in our ARC on 1 July 2014 (see Table 1). The immigration office bases its decisions on availability of the treatment in the country even if accessible only to a limited number of patients.

**Conclusions:**

Decisions taken by the Belgian authorities for the last two years concerning HIV-infected asylum seekers do not guarantee the continuity of care of those patients and push them towards illegality. Such decisions ignore the international commitments of Belgium in the fight against HIV [2] and are contradictory with the recommendations of the recent resolution of the Council of Europe [1]. An approach more respectful of Human Rights in the decisions concerning the seropositive asylum seekers patients taken by the authorities is urgently needed in Belgium. We invite our European colleagues to describe the situation of the HIV asylum seekers in their countries.

**Table 1:** Characteristics and detailed situations of the HIV positive asylum seekers in the ARC of Charleroi

Country of origin (N = 45) (n)	Rwanda (7), Democratic Republic of Congo (6), other country of sub-Saharan Africa (23), Maghreb (2), ex USSR (5), other (2)
Reported reason for migration of the patients ignoring their HIV seropositivity or positive status (N = 37); (n (%))	Political (18 (47%)), personal threats (5 (11%)), health (1 (11%)), economical (3 (7%)), familial (1 (4%)), unknown (9 (20%))
Reported reason for migration among patients knowing their HIV positive status (N = 8); (n)	Health (4 (multiR virus (2), treatment unaffordable (1), hemodialysis needed (1)) political threat (3), family reunification (1)
Reason for regularization (N = 4); (n)	Political reason (2), family reunification (1), humanitarian reason (1). No regularization through the medical procedure
Retention in care (N = 45) (n (%))	LFU (12 (27%)), followed in another ARC (8 (18%)), back to their home country (2 (4%)), follow up in our ARC (23 (51%))
